# Environment impacts sexual preference: dopamine sends signals under pressure to decide which sex you like

**DOI:** 10.1038/s41392-025-02211-0

**Published:** 2025-04-18

**Authors:** Rou Li, Lin Liu, Min Wu

**Affiliations:** 1 Medical College, Guizhou University, Guiyang, Guizhou China; 2 Department of Respiratory and Critical Care Medicine, NHC Key Laboratory of Pulmonary Immunological Diseases, Guizhou Provincial People’s Hospital, Guiyang, Guizhou China; 3 Wenzhou Institute, University of Chinese Academy of Sciences, Wenzhou, Zhejiang China

**Keywords:** Molecular neuroscience, Neurogenesis

**Fig. 1:**
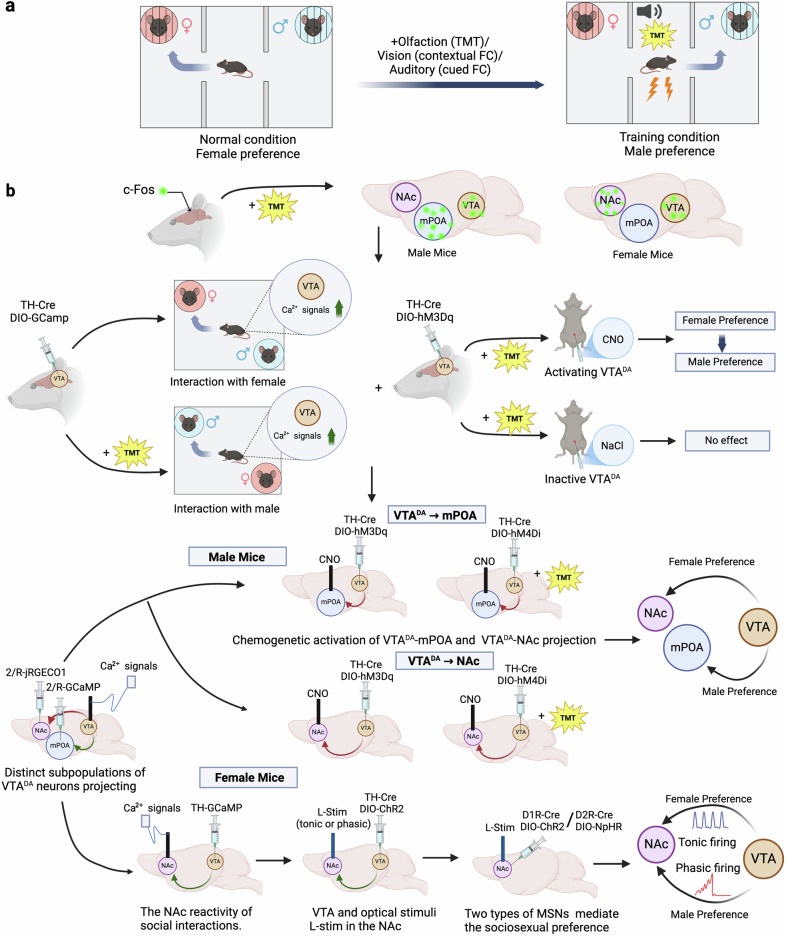
**a** Schematic diagram illustrating the social preference test. Both male and female mice exhibited a preference for social interaction with females. However, this preference shifted to male preference when facing survival threats (TMT, contextual FC, or cued FC). **b** The sexually dimorphic DA circuits involve the sociosexual preferences of females and males. C-Fos staining revealed that the activity changes in VTA^DA^ and their downstream circuits, the mPOA and the NAc, are closely related to these changes. A calcium signaling fiber optic recording method for real-time monitoring of VTA^DA^ neural activity during social processes, along with a chemical genetics approach for activating VTA^DA^ neurons. Dual-color calcium signal fiber recording combined with viral tracing for synchronous monitoring of VTADA neuronal subpopulation activity. Chemical genetic methods were employed to manipulate the VTA^DA^-mPOA and VTA^DA^-NAc projection. The competitive balance between VTA^DA^-NAc and the defense loop VTA^DA^-mPOA determines the social preferences of male animals. By employing optogenetic techniques, the VTA-NAc projection was stimulated with light in both tonic and phasic discharge modes. Female individuals selectively regulate downstream neurons by modulating firing patterns in the VTA^DA^-NAC) ultimately shaping their social preferences. TMT trimethylthiazoline, FC fear conditioning, VTA ventral tegmental area, mPOA midbrain preoptic area, NAc nucleus accumbens, CNO clozapine N-oxide, D1R type 1 DA receptor, D2R type 2 DA receptor. This figure was created with BioRender.com

A study by Wei et al. in *Science* identified a unique sexual binary neural circuit encoding a sexual preference switch, known as the ventral tegmental area dopaminergic (VTA^DA^) circuit, which exhibits a preference for female social interactions among both sex mice, but switches to male preferences when confronted with survival threats (Fig. 1).^1^

Innate social behavior is essential for individuals to acquire external resources and respond to survival threats. Regarding social behavior, the sex of social partners is a significant factor impacting social decision-making.^2^ Opposite-sex interactions support mating and species continuity, while same-sex interactions enhance health, emotional support, and resource access. However, the neural mechanisms behind sex preference in social activities, particularly under environmental pressure, remain unclear. Wei et al. conducted a three-chamber social test and discovered that both male and female mice exhibited a preference for social interaction with females. However, under life-threatening conditions, this preference shifted markedly toward males, mediated through multiple sensory pathways. These findings suggest that social decision-making is integrated process responses to both innate needs and external environmental factors (Fig. 1a).

Next, the authors employed c-Fos staining to elucidate the neural circuit. In males, changes in neural activity were observed in dopamine (DA) neurons and their downstream midbrain preoptic area (mPOA), associated with defense, mating, and paternal behavior. In females, changes occurred in DA neurons and the nucleus accumbens (NAc), the brain’s reward center. The authors applied calcium signal fiber optic recording to monitor the VTA^DA^ neural activity in real-time during social interactions, discovering that both male and female mice exhibit heightened DA neuron excitability when interacting with females. However, under teimethylthiazoline (TMT) conditions, DA neuron excitability associated with male social interactions is significantly heightened. Furthermore, stimulating VTA^DA^ neurons shifts the preference, suggesting that the VTA^DA^ system may serve as the decision-making center for social preferences (Fig. 1b).

To affirm the role of the DA system, virus tracing and dual-color calcium signaling recordings revealed in male mice activating VTA-mPOA defense circuit DA neurons during same-sex interactions and VTA-NAc reward pathway DA neurons during opposite-sex interactions. Notably, no such VTA-mPOA circuit correlation was observed in female mice. Chemogenetic activation of the VTA^DA^-mPOA circuit enhances male preference, while its inhibition reverses stress-induced male animals’ preference for same-sex behavior. Conversely, activating the VTA^DA^-NAc reward reverses male preference facing challenges, whereas its inhibition suppresses female preference in males. Optogenetic stimulation of the VTA^DA^-NAc projection stimulated that phasic firing enhanced female preference in females, while tonic firing shifted preference from females to males (Fig. 1b). Meanwhile, phasic firing-like optogenetic triggered rapid, high-concentration DA release, activating type 1 DA receptor (D1R) neurons, and promoting female social preference. Additionally, tonic firing-like excitation induced sustained low-concentration DA release, enhancing type 2 DA receptor (D2R) neuron inhibition and promoting male social preference, indicating that the balance between VTA^DA^-NAc and VTA^DA^-mPOA pathways determines the social preferences of male mice, while female sex choice is impacted by the VTA^DA^-NAc firing patterns.

Dopamine circuits are highly conserved throughout evolution, regulating reward, emotion, social behavior, and movement, with dysfunction linked to conditions like anxiety, depression, lack of motivation, and Parkinson’s disease. The dopamine reward system serves as a critical hub for integrating external positive and negative stimuli and projects extensively to key brain regions, regulating diverse physiological functions. This study highlights the DA system’s essential role in encoding social decision-making and reveals its sexually dimorphic nature in mediating social sexual preferences.

In Wei’s study, non-estrous female mice were used but lacked detailed descriptions of the animal models. Research showed that VTA^DA^ activity varies throughout the estrous cycle in rodents, which may be related to estradiol variations, subsequently affecting neuronal excitability and stress responses. Under stress, the estrous cycle and estrogen signaling pathways modify the physiological functions of DA neurons, resulting in behavioral differences.^3^ The estrus cycle can be monitored via vaginal cell collection (swabs or lavage) and analysis of cell type and morphology, or hormonal interference can be eliminated through ovariectomy. Misiołek et al. evaluated an average phase over four days for pretest-posttest and inter-sex comparisons to mitigate estrous cycle effects on sex-based differences.^4^ Social behavior is influenced by hormones, environmental factors, physiological processes, and other elements beyond neural circuits. The interactions among various neural regulatory systems impact social behavior in intricate ways.^5^ Considering these technical complexities and challenges, future research must use approximate models and preferably human subjects to clarify dimorphic DA circuits underlying sexual preference.

Although this study yielded significant findings, numerous issues emerge and require further detailed investigation. For instance, it is essential to understand how various sensory inputs converge into specific subpopulations of neurons and how the firing patterns are precisely regulated by external survival pressures. Additionally, exploring detailed neural circuit mechanisms, such as the influence of sex hormones on gender-related social decision-making, is essential. The roles of NAC and mPOA projections in non-human primates and humans, as well as their contributions to broader social behaviors (e.g., social avoidance, support, and hierarchy), also require examination. The observed gender-dimorphic social preference in rodents may provide insights into human social behavior, necessitating validation in non-human primates or humans to determine evolutionary conservation or divergence. This research will enhance our understanding of the functional and adaptive roles of neural circuits across species. While it explores the immediate effects of survival threats on social preferences, whether these environmental factors induce long-term neuroplastic changes remains elusive. For instance, repeated exposure to survival threats may irreversibly alter sex-specific dopamine circuits, potentially affecting long-term social behavior and mental health. Investigating how sexual preferences are influenced by other physiological or environmental factors (e.g., extreme climate change, and social stress) will further elucidate the neural mechanisms underlying social behavior.

In summary, this work unveils that both sexes exhibit female preferences under physiological conditions; however, under survival threats, their preferences shift to males. Males determine social preferences through the competitive balance of VTA^DA^-mPOA and VTA^DA^-NAc projections, while females rely on the modulation of discharge modes within the VTA^DA^-NAc projections. Despite the designing of experiments being unperfect, this study offers a new perspective for understanding the emotional and behavioral differences between males and females, representing theoretical basis for sex-specific treatments of related neuronal disorders.
